# Preterm birth and small for gestational age potentiate the association between maternal hypertensive pregnancy and childhood autism spectrum disorder

**DOI:** 10.1038/s41598-023-36787-w

**Published:** 2023-06-13

**Authors:** Lan-Wan Wang, Hung-Chih Lin, Ming-Luen Tsai, Yu-Tzu Chang, Yu-Chia Chang

**Affiliations:** 1 Department of Pediatrics, Chi Mei Medical Center, #901, Chung Hwa Rd., Yung Kang District, Tainan, 710402 Taiwan; 2 Department of Biotechnology and Food Technology, Southern Taiwan University of Science and Technology, Tainan, Taiwan; 3 Department of Pediatrics, China Medical University Children’s Hospital, China Medical University, Taichung, Taiwan; 4 Department of Pediatrics, Asia University Hospital, Asia University, Taichung, Taiwan; 5 School of Post-Baccalaureate Chinese Medicine, China Medical University, Taichung, Taiwan; 6 Department of Long-Term Care, College of Health and Nursing, National Quemoy University, #1, University Rd., Jinning Township, Kinmen County, 892009 Kinmen, Taiwan; 7 Department of Healthcare Administration, College of Medical and Health Science, Asia University, Taichung, Taiwan

**Keywords:** Paediatric research, Autism spectrum disorders, Hypertension

## Abstract

Children of mothers with hypertensive disorders of pregnancy (HDP) have high rates of preterm-birth (gestational age  < 37 weeks) and small-for-gestational-age (SGA), both of which are risk factors of autism spectrum disorder (ASD). This study tested the multiple-hit hypothesis that preterm-birth and SGA in the neonatal period might potentiate the antenatal impact of HDP to increase childhood ASD hazards, and HDP might not be a major contributor. The propensity-score-matched cohort enrolled 18,131 mother–child pairs with HDP and 90,655 normotensive controls between 2004 and 2011. Children with siblings born to the same mothers were excluded for analysis to reduce the potential familial-genetic influence. HDP were classified into chronic-hypertension, gestational-hypertension, preeclampsia, and preeclampsia-with-chronic-hypertension. Using the normotensive group as the reference, the associations between HDP subgroups and the cumulative ASD risks were assessed with hazard ratios, and the effects of preterm-birth and SGA on the associations were examined. The HDP group had a higher cumulative rate of ASD (1.5%) than the normotensive group (1.2%). Preterm-birth and SGA exerted moderating effects to aggravate ASD hazards in children exposed to chronic-hypertension or gestational-hypertension. None of HDP types significantly contributed to ASD after adjustments. In conclusion, antenatal HDP exposure might predispose to ASD outcome through susceptibility to the impact of preterm-birth and SGA.

## Introduction

Autism spectrum disorder (ASD) is a neurodevelopmental disorder characterized by impairment in social interaction, communication deficits and stereotyped behavior^1,2^, and occurs in approximately 1–2% of children^3,4^. Children with ASD may have not only behavioral problems and social incompetence requiring early intervention programs and special education, but also higher rates of comorbid medical conditions, such as intellectual disability and attention deficit hyperactivity disorder^4,5^. The co-morbidities in children with ASD may further negatively impact their mental health and social adaptation, leading to increased medical, educational and social expenses for special health care needs.

The Developmental Origins of Health and Disease hypothesis suggests that prenatal events and maternal well-being during pregnancy may have long-lasting effects on children’s neurodevelopment^6^. Among the prenatal events, hypertensive disorders of pregnancy (HDP), such as chronic hypertension or preeclampsia, complicate approximately 5–10% of all pregnancies leading to maternal and fetal morbidity and mortality^7^. As there have been increased trends of HDP and ASD during the past decades^8,9^, it is important to investigate whether HDP exert maternal–fetal influence upon offspring’s ASD outcome.

Although meta-analysis studies have shown that children exposed to HDP, particularly preeclampsia, may have higher risk of ASD, it remains unclear whether the association is causal or attributed to residual or unmeasured confounding^10^. Children born to mothers with HDP have high rates of preterm birth (gestational age [GA] ≤ 36 weeks) and intrauterine growth restriction/small for gestational age (SGA)^11,12^, both of which are well-known risk factors of ASD^13,14^. Previous studies have reported children exposed to both preeclampsia and preterm birth/SGA had increased risks of ASD than those exposed to preeclampsia alone^15,16^, and the association between preeclampsia and ASD might be mediated through preterm birth or SGA^15,17,18^. There may be multiple hits of insults during the process leading to neuropsychiatric disorders^19^, and antenatal exposure to HDP may make the developing brain more susceptible to subsequent injuries associated with preterm birth and SGA in the perinatal and neonatal period. It remains unclear whether preterm birth and SGA may aggravate the impact of maternal HDP in early life to increase the risk of childhood ASD, and whether different types of HDP have independent contribution to ASD hazards under the confounding influence of preterm birth and SGA. Understanding the interrelationship between antenatal HDP exposure and the following preterm birth/SGA may help strategy making to reduce ASD hazards.

Using a propensity-score-matched cohort from the nationwide registry of mother–child database in Taiwan, this study tested the multiple-hit hypothesis that HDP might not be a major contributor for ASD outcome, and preterm birth and SGA in the neonatal period might potentiate the antenatal impact of HDP to increase ASD hazards in childhood.

## Results

### Characteristics of mother–child pairs in the normotensive and HDP groups

There were 18,131 children born to mothers with HDP, including 5488 (30.3%) chronic hypertension, 3204 (17.7%) gestational hypertension, 7673 (42.3%) preeclampsia, and 1766 (9.7%) preeclampsia with superimposed chronic hypertension. The comparisons between the HDP and normotensive group are shown in Table 1. Compared to the normotensive group, the HDP group had higher rates of pre-pregnancy obesity (0.8% vs 0.1%), pre-pregnancy or gestational diabetes mellitus (DM, 16.8% vs. 7.2%), and maternal psychiatric/mental disorders (6.6% vs. 4.4%); and lower rates of perinatal infection (1.9% vs. 3.0%) and antepartum hemorrhage (1.0% vs. 1.4%), especially the subgroups of chronic hypertension with or without preeclampsia. Although the HDP and normotensive group did not differ in the mean values and distribution of GA, the HDP group had significantly higher rate of SGA (22.9% vs. 9.7%, *p* < 0.001) with low birth weights < 2500 grams (27.3% vs. 13.8%, *p* < 0.001), and higher incidence of neonatal intensive care unit (NICU) admission (0.9% vs. 0.5%, *p* < 0.001) than the normotensive group. Children in the HDP subgroups of preeclampsia with or without superimposed chronic hypertension had higher rates of preterm birth (GA ≤ 36 weeks, 33–42%) and SGA (26–28%) than those in the subgroups of chronic hypertension and gestational hypertension (preterm birth 17–22%; SGA 17–19%) (Table 1).

**Table 1:** Characteristics of mother–child pairs in the groups of normotension and hypertensive disorders of pregnancy (HDP).

	Normotension (n = 90,655)	HDP (n = 18,131)	HDP subgroup				*p* value (HDP vs. normotension)
			Chronic hypertension (n = 5488)	Gestational hypertension (n = 3204)	Preeclampsia (n = 7673)	Preeclampsia with chronic hypertension (n = 1766)	
Maternal data							
Delivery age, year, mean (SD)	31.6 (5.1)	31.8 (5.1)	33.0 (5.2)	31.2 (5.0)	31.0 (5.0)	32.3 (4.9)	1.00
Cesarean section	59,990 (66.2)	11,999 (66.2)	3432 (62.5)	1632 (50.9)	5534 (72.1)	1401 (79.3)	0.99
Pre-pregnancy obesity	68 (0.1)	143 (0.8)	78 (1.4)	10 (0.3)	32 (0.4)	23 (1.3)	< 0.001
Diabetes mellitus	6532 (7.2)	3048 (16.8)	1157 (21.1)	512 (16.0)	997 (13.0)	382 (21.6)	< 0.001
Psychiatric/mental illness	4026 (4.4)	1196 (6.6)	607 (11.1)	120 (3.7)	335 (4.4)	134 (7.6)	< 0.001
Perinatal infection	2698 (3.0)	343 (1.9)	102 (1.9)	69 (2.2)	146 (1.9)	26 (1.5)	< 0.001
Antepartum hemorrhage	1243 (1.4)	176 (1.0)	46 (0.8)	25 (0.8)	87 (1.1)	18 (1.0)	< 0.001
Child data							
Gestational age, week							0.90
> 36	65,685 (72.5)	13,137 (72.5)	4306 (78.5)	2656 (82.9)	5154 (67.2)	1021 (57.8)	
32–36	22,478 (24.8)	4485 (24.7)	1061 (19.3)	515 (16.1)	2274 (29.6)	635 (36)	
< 32	2492 (2.8)	509 (2.8)	121 (2.2)	33 (1.0)	245 (3.2)	110 (6.2)	
Mean (SD)	37.5 (2.3)	37.2 (2.3)	37.4 (2.1)	37.8 (1.9)	37.0 (2.4)	36.3 (2.7)	
Birth weight, gram							< 0.001
> 2499	78,192 (86.3)	13,185 (72.7)	4487 (81.8)	2642 (82.5)	5003 (65.2)	1053 (59.6)	
1500–2499	10,578 (11.7)	4095 (22.6)	850 (15.5)	513 (16.0)	2189 (28.5)	543 (30.7)	
< 1500	1885 (2.1)	851 (4.7)	151 (2.8)	49 (1.5)	481 (6.3)	170 (9.6)	
Mean (SD)	2986 (550)	2823 (697)	2963 (629)	2973 (585)	2711 (732)	2595 (773)	
Small for gestational age	8784 (9.7)	4163 (22.9)	937 (17.1)	605 (18.9)	2156 (28.1)	465 (26.3)	< 0.001
Male	46,241 (51.0)	9242 (50.9)	2881 (52.5)	1612 (50.3)	3844 (50.1)	905 (51.3)	0.93
NICU admission	449 (0.5)	166 (0.9)	36 (0.7)	17 (0.5)	88 (1.1)	25 (1.4)	< 0.001
Autism spectrum disorder	1054 (1.2)	269 (1.5)	78 (1.4)	48 (1.5)	112 (1.5)	31 (1.8)	< 0.001

Data shown as n (%) unless otherwise indicated.

*SD* standard deviation, *NICU* neonatal intensive care unit.

### Cumulative incidences of ASD in the normotensive and HDP groups

**Figure 1:**
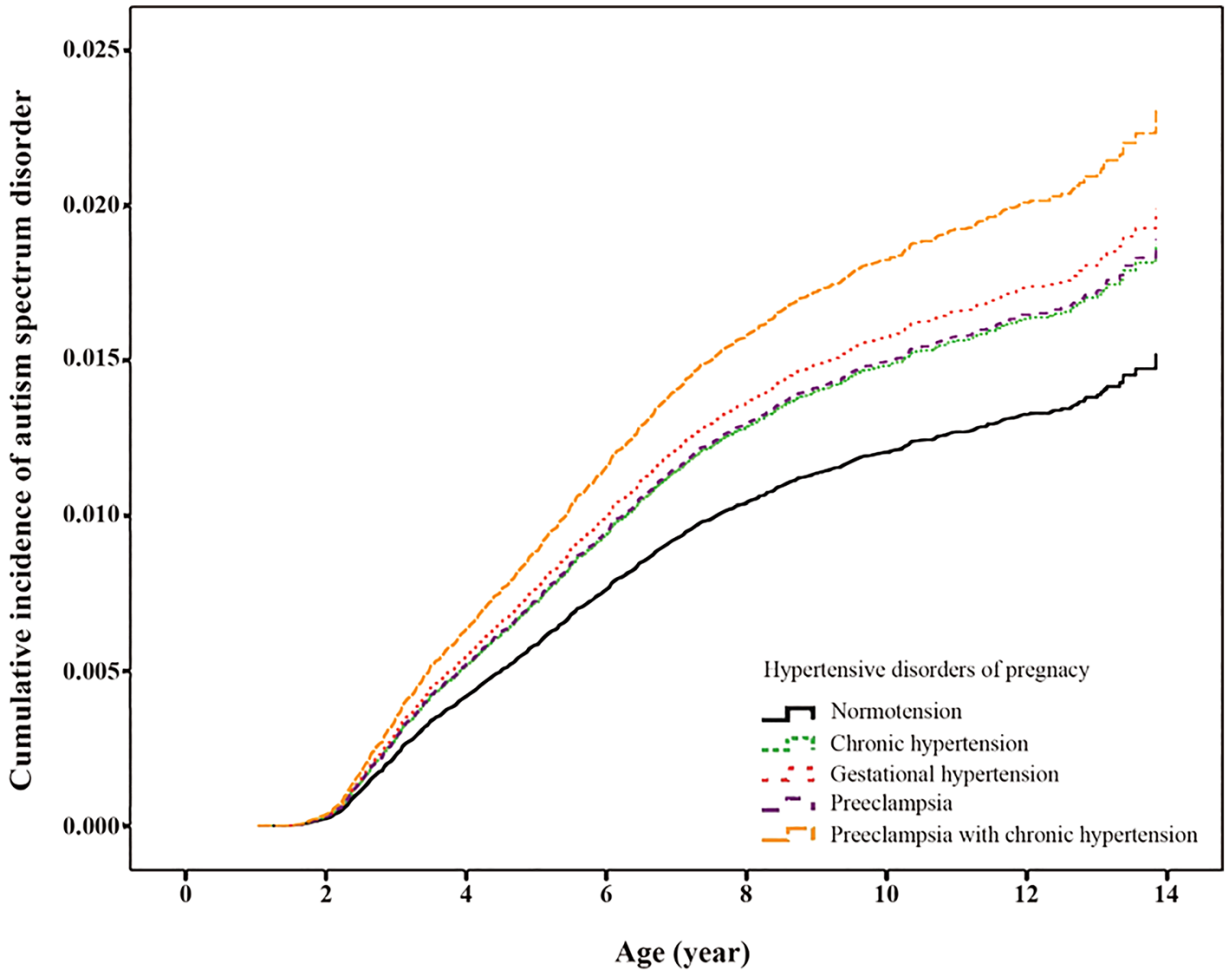
Crude cumulative incidences of autism spectrum disorder by the age of 14 years among children of mothers without hypertension (normotension) and with different types of hypertensive disorders of pregnancy.

There were 1323 (1.2%) children who had ASD in the study cohort, with a higher rate in the HDP than in the normotensive group (1.5% vs. 1.2%, *p* < 0.001). Among children exposed to maternal HDP, the crude cumulative incidences of ASD by 14 years of age were highest for preeclampsia with superimposed chronic hypertension (1.92%, 95% confidence interval 1.30–2.74), followed by gestational hypertension (1.59%, 1.18–2.12), preeclampsia (1.45%, 1.15–1.81), and chronic hypertension (1.44%, 1.18–1.74), all of which were higher than those in the unexposed children (1.20%, 1.13–1.27) (Fig. 1).

### Potential predictors of ASD hazards in univariable analysis

Using the normotensive group as the reference, children exposed to maternal preeclampsia with superimposed chronic hypertension had the highest cumulative risk (or hazard) of ASD (hazard ratio 1.52, 95% confidence interval 1.06–2.17), followed by those exposed to preeclampsia alone (1.24, 1.02–1.51); whereas children exposed to maternal chronic hypertension (1.23, 0.98–1.55) or gestational hypertension (1.31, 0.98–1.75) had no significantly increased hazards (Table 2). Other significant maternal predictors of ASD included higher maternal age (per year) at delivery (1.04, 1.03–1.05), pre-pregnancy obesity (3.25, 1.63–6.52), DM (1.29, 1.09–1.53), psychiatric/mental disorders (1.65, 1.35–2.02), perinatal infection (1.35, 1.01–1.79), antepartum hemorrhage (1.70, 1.17–2.47), and cesarean section (1.17, 1.04–1.31). GA and birth weights were inversely associated with cumulative ASD risks, with the highest hazards in children born at GA < 32 weeks (2.87, 2.30–3.58) or with birth weights < 1500 grams (2.74, 2.20–3.41). Children who were male (4.83, 4.18–5.59), SGA (1.51, 1.31–1.75), and born in summers (1.35, 1.16–1.57) or in the last 3 enrollment years (2009–2011, hazard ratio 1.40–1.78) also had higher hazards of ASD (Table 2).

**Table 2:** Potential predictors of autism spectrum disorder in univariable models.

Variable	Cumulative incidence and risk of ASD			*p* value
	n/N	%	Hazard ratio (95% CI)	
Maternal factors				
Age at delivery, per year	–	–	1.04 (1.03–1.05)	< 0.001
Hypertensive disorders of pregnancy				
Chronic hypertension	78/5488	1.4	1.23 (0.98–1.55)	0.08
Gestational hypertension	48/3204	1.5	1.31 (0.98–1.75)	0.07
Preeclampsia	112/7673	1.5	1.24 (1.02–1.51)	0.03
Preeclampsia with chronic hypertension	31/1766	1.8	1.52 (1.06–2.17)	0.02
Pre-pregnancy obesity	8/211	3.8	3.25 (1.63–6.52)	< 0.001
Diabetes mellitus	144/9580	1.5	1.29 (1.09–1.53)	0.01
Psychiatric/mental illness	101/5222	1.9	1.65 (1.35–2.02)	< 0.001
Perinatal infection	49/3041	1.6	1.35 (1.01–1.79)	0.04
Antepartum hemorrhage	28/1419	2.0	1.70 (1.17–2.47)	0.01
Cesarean section	922/71,989	1.3	1.17 (1.04–1.31)	0.01
Infant factors				
Preterm birth < 37 weeks				
32–36	383/26,963	1.4	1.46 (1.27–1.68)	< 0.001
< 32	88/3001	2.9	2.87 (2.30–3.58)	< 0.001
Low birth weight < 2500 g				
1500–2499	235/14,673	1.6	1.31 (1.16–1.48)	< 0.001
< 1500	85/2736	3.1	2.74 (2.20–3.41)	< 0.001
Small for gestational age	224/12,947	1.7	1.51 (1.31–1.75)	< 0.001
Male sex	1102/55,483	2.0	4.83 (4.18–5.59)	< 0.001
Birth season				
Winter	294/26,385	1.1	1.00 (reference)	
Spring	301/25,430	1.2	1.07 (0.91–1.25)	0.44
Summer	400/27,323	1.5	1.35 (1.16–1.57)	< 0.001
Autumn	328/29,648	1.1	1.04 (0.89–1.22)	0.64
Birth year				
2004	155/12,288	1.3	1.00 (reference)	
2005	163/12,666	1.3	1.12 (0.89–1.40)	0.34
2006	157/13,518	1.2	1.05 (0.84–1.32)	0.65
2007	149/13,518	1.1	1.05 (0.84–1.33)	0.66
2008	143/13,848	1.0	1.05 (0.83–1.33)	0.68
2009	186/14,754	1.3	1.40 (1.12–1.74)	< 0.001
2010	180/12,546	1.4	1.78 (1.42–2.23)	< 0.001
2011	190/15,648	1.2	1.76 (1.41–2.21)	< 0.001
Neonatal intensive care unit admission	10/615	1.6	1.36 (0.73–2.53)	0.34

*ASD* autism spectrum disorder, *CI* confidence interval.

### Effects of preterm birth and SGA on the association between HDP and ASD

**Figure 2:**
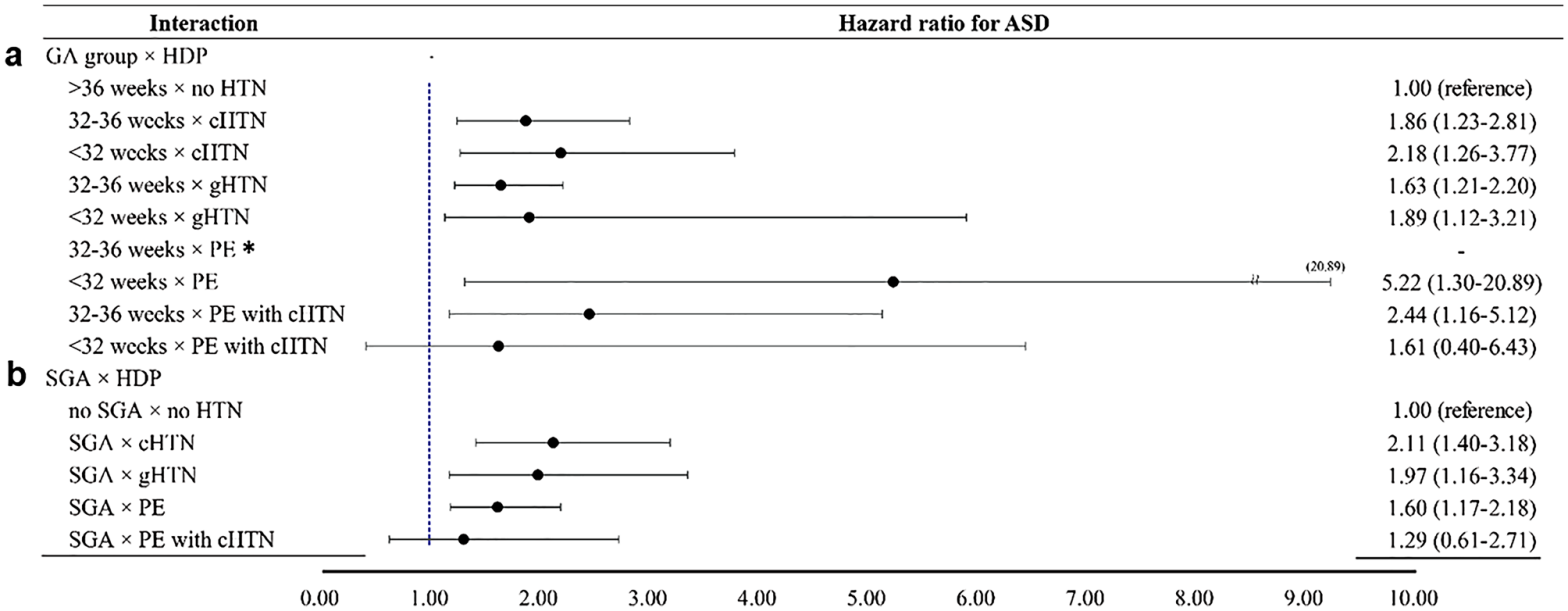
Moderating effects of preterm birth and small for gestational age (SGA) on the association between hypertensive disorders of pregnancy (HDP) and autism spectrum disorder (ASD) hazards. HDP included chronic hypertension (cHTN), gestational hypertension (gHTN), preeclampsia (PE), and preeclampsia with superimposed chronic hypertension (PE with cHTN). The gestational ages (GA) were classified into > 36, 32–36 and < 32 weeks. (**a**) The interactions between preterm birth (GA 32–36, < 32 weeks) and different HDP on ASD hazard ratios, using children born at GA > 36 weeks (term birth) without maternal hypertension (HTN) as the reference. (**b**) The interactions between SGA and different HDP on ASD hazard ratios, using children born without SGA and without maternal hypertension as the reference. *No ASD occurrence in the subgroup of PE and GA 32–36 weeks.

We then examined the potential effects of preterm birth (GA ≤ 36 weeks) and SGA on the association between different HDP types and ASD. Using the groups of normotension and GA > 36 weeks as the reference, preterm birth had little mediating influence (Supplementary Table S1) and predominantly exerted moderating effects, which considerably increased ASD hazards of chronic hypertension and gestational hypertension as GA decreased from 32–36 weeks to < 32 weeks (Fig. 2a). Using the groups of normotension and non-SGA as the reference, SGA also had more moderating than mediating effects (Supplementary Table S1), with substantial increases of ASD hazards for all HDP types except for preeclampsia with superimposed chronic hypertension (Fig. 2b).

### HDP types, preterm birth and SGA in multivariable models for ASD hazards

To determine whether different types of maternal HDP independently contributed to the cumulative risks of ASD, the effects of HDP subgroups were examined in multivariable models using the normotensive group as the reference. We first included HDP subgroups and GA group (< 32, 32–36, > 36 weeks) in Model 1, and then incorporated Model 1 and SGA in Model 2, and finally included Model 2 and other covariates in Model 3 (Table 3). Before adjustments, preeclampsia alone or with superimposed chronic hypertension had substantial contribution to ASD outcome. After adjusting for GA group using GA > 36 weeks as the reference in Model 1, preeclampsia alone remained significant (adjusted hazard ratio 1.22, 95% confidence interval 1.01–1.48; *p* = 0.04), while preeclampsia with superimposed chronic hypertension became insignificant (1.40, 0.98–2.00). After further adjusting for SGA in Model 2, the significant effect of preeclampsia disappeared (1.11, 0.91–1.36). In Model 3 that included all covariates, none of any HDP had significant contribution to ASD outcome, while both the effects of preterm birth and SGA remained significant in Model 3 with high hazards for children born at GA < 32 weeks (2.48, 1.97–3.13; *p* < 0.001) or being SGA (1.51, 1.31–1.75;* p* < 0.001). For each HDP subgroup, the adjusted hazard ratio in the final Model 3 was less than the adjusted odds ratio estimated by E-value sensitivity analysis (Table 4), supporting the predictive accuracy for ASD outcome.

**Table 3:** Association between different types of maternal hypertensive disorders of pregnancy and childhood autism spectrum disorder hazards in multivariable models.

Variable	Autism spectrum disorder hazard ratio (95% confidence interval)			
	Unadjusted	Adjusted		
		Model 1	Model 2	Model 3
Hypertensive disorders of pregnancy				
No	1.00 (reference)	1.00 (reference)	1.00 (reference)	1.00 (reference)
Chronic hypertension	1.23 (0.98–1.55)	1.26 (0.99–1.59)	1.22 (0.97–1.53)	1.04 (0.83–1.32)
Gestational hypertension	1.31 (0.98–1.75)	1.38 (1.03–1.84)	1.32 (0.98–1.76)	1.32 (0.99–1.77)
Preeclampsia	1.24 (1.02–1.51)	1.22 (1.01–1.48)	1.11 (0.91–1.36)	1.15 (0.95–1.41)
Preeclampsia with chronic hypertension	1.52 (1.06–2.17)	1.40 (0.98–2.00)	1.29 (0.90–1.85)	1.18 (0.82–1.70)
Gestational age, week				
> 36	1.00 (reference)	1.00 (reference)	1.00 (reference)	1.00 (reference)
32–36	1.31 (1.16–1.48)	1.31 (1.16–1.48)	1.33 (1.17–1.50)	1.27 (1.12–1.43)
< 32	2.74 (2.20–3.41)	2.74 (2.20–3.41)	2.80 (2.25–3.49)	2.48 (1.97–3.13)
Small for gestational age				
No	1.00 (reference)	–	1.00 (reference)	1.00 (reference)
Yes	1.51 (1.31–1.75)	–	1.51 (1.30–1.75)	1.51 (1.31–1.75)

Model 1 included hypertensive disorders of pregnancy and gestational age group; Model 2 included Model 1 and small for gestational age; Model 3 included Model 2, maternal factors (age at delivery, pre-pregnancy obesity, diabetes mellitus, psychiatric/mental illness, perinatal infection, antepartum hemorrhage, cesarean section), and infant factors (male sex, birth season, birth year).

**Table 4:** Sensitivity analysis of unmeasured confounding for autism spectrum disorder outcome.

Hypertensive disorders of pregnancy	E-value analysis for autism spectrum disorder outcome	
	Adjusted odds ratio^a^	Lower confidence limit^b^
Chronic hypertension	1.24	NA
Gestational hypertension	1.97	NA
Preeclampsia	1.57	NA
Preeclampsia with chronic hypertension	1.64	NA

^a^Indicates the minimum association that the confounders would have to have with both exposure and outcome to explain away the observed adjusted hazard ratios.

^b^Indicates the minimum association that confounders would have to have with both exposure and outcome to move the lower confidence limit to include the null. Not applicable (NA) indicates the lower confidence limit starts below 1.00.

## Discussion

This propensity-score-matched cohort study investigated cumulative ASD risks in children born to mothers with different types of HDP, and determined the confounding effects of preterm birth and SGA which were highly associated with both HDP and ASD. We found that preterm birth and SGA not only had significant contribution to ASD outcome, but also potentiated the association between HDP and ASD. Preterm birth, particularly GA < 32 weeks, substantially increased ASD risks in children exposed to chronic hypertension or gestational hypertension, while SGA raised ASD hazards in children of mothers with chronic hypertension, gestational hypertension, or preeclampsia. Although children of preeclamptic mothers with or without chronic hypertension had increased ASD hazards, the causal associations were greatly attenuated after adjusting for preterm birth and SGA.

Previous studies examining the association between maternal HDP and childhood ASD often focused on preeclampsia^15,17,18,20–22^. Most population-based cohort studies found children exposed to maternal preeclampsia had higher hazards of ASD (adjusted hazard ratios ranged from 1.27 to 1.87) than those without exposure^17,18,20^, and early-onset or severe preeclampsia further increased the risks^17^. A few case–control studies showed children with ASD had higher rates of maternal preeclampsia than those with typical development, and preeclampsia had significant contribution to ASD (adjusted odds ratio ranged from 1.69 to 2.36)^21,22^. Recent studies have examined the confounding influence of familial genetic factors using the sibling pair analysis. After further adjusting for shared familial factors, two studies demonstrated preeclampsia or unclassified HDP was associated with ASD^17,23^, while another study did not^15^. Although most studies favored the association between preeclampsia and ASD, some important confounding factors may not always be considered or available to justify the causal relationship^10^. After reducing the potential sibling effects, we found that maternal preeclampsia had no significant association with ASD outcome after adjusting for preterm birth and SGA, and the findings were supported by the sensitivity analysis of unmeasured confounding.

Few studies investigated ASD outcomes in children exposed to maternal HDP other than preeclampsia. One study showed children born to mothers with chronic or gestational hypertension had increased risks of ASD^17^, while other studies did not^18,24^. When preeclampsia was superimposed on chronic hypertension, one study reported a higher risk of ASD in children exposed to both (adjusted hazard ratio 1.48, 95% confidence interval 1.21–1.80) than in those exposed to chronic hypertension (1.10, 0.98–1.24) or preeclampsia (1.36, 1.30–1.42) only^17^. Our study found no increased ASD hazards in children exposed to maternal chronic or gestational hypertension, and preeclampsia superimposed on chronic hypertension did not further increase ASD risk (1.18, 0.82–1.70) than either preeclampsia (1.15, 0.95–1.41) or chronic hypertension (1.04, 0.83–1.32) alone.

Our study demonstrated that preterm birth and SGA exerted moderating effects to strengthen the association between HDP and ASD, implicating there might be multiple hits rather than single HDP exposure in the process leading to ASD outcome. Maternal hypertension during the pregnancy, particularly preeclampsia, may negatively affect the fetal environment by triggering insults such as uteroplacental hypoperfusion, oxidative stress, placental inflammation and nutrient insufficiency, resulting in intrauterine growth restriction/SGA or preterm delivery^11,12,25^. The antenatal detrimental events may sensitize the developing brain, making it more vulnerable to injuries from subsequent insults such as preterm birth and SGA^26^. Infants born very preterm often experience intertwining events of hypoxia/ischemia and infection in the perinatal and neonatal periods, which might have cumulative effects on the risk of neurodevelopmental impairment^27^. The higher rates of perinatal asphyxia and neonatal hypoglycemia in SGA infants might also predispose to unfavorable neurodevelopmental outcomes^28^. The early-life adverse events from the prenatal to neonatal period may not only damage the brain but also affect brain development through epigenetic modification of brain structure and function, leading to increased hazards of neuropsychiatric disorders^29^.

Using a large cohort of mother–child pairs with well-matched covariates in the inter-group backgrounds, this study demonstrated the potentiating effects of preterm birth and SGA on the association between maternal HDP and childhood ASD. The study cohort constructed by propensity score matching is not applicable for examining the potential familial genetic influence on ASD hazards. We therefore did not include children with siblings born to the same mothers for analysis in the propensity-score-matched cohort. The generalizability of our findings may be validated in another population-based cohort studies.

In conclusion, our findings supported the multiple-hit hypothesis that preterm birth and SGA in the neonatal period might potentiate the antenatal impact of HDP to increase ASD hazards in childhood. The associations between HDP and ASD were greatly attenuated after adjusting for preterm birth and SGA, suggesting that HDP might serve as a sensitizer rather than a major hitter on the developing brain. Adequate neonatal care for preterm and SGA infants and antenatal blood pressure control for HDP mothers, may help reduce the risks and complications of preterm delivery and SGA to diminish ASD hazards in offspring.

## Methods

### Data sources

This cohort study used the Maternal and Child Health Database (MCHD) which link the data of offspring and their parents by integrating three nationwide databases in Taiwan, including Birth Registration Database, National Register of Death, and National Health Insurance Research Database (NHIRD). The MCHD contains perinatal records on 99.8% of all births in Taiwan from 1st January 2004 to 31st December 2011, and the data of medical claims and diagnoses during hospitalization and outpatient visits for mothers from 1998 to 2011, and for children from 2004 to 2011^30^. We first obtained the mother–child data with birth registration between 2004 and 2011 from MCHD, and followed up these children until 31st December 2017 when they were at least 6 years old (range 6–13 years). The children’s data from 2012 to 2017 were extracted from NHIRD, which contains the medical claims data of more than 99.9% of the population in Taiwan covered by National Health Insurance Program^31^. Medical diagnoses were coded using the International Classification of Diseases, Ninth Revision (ICD-9) from 1998 to 2015, and International Statistical Classification of Diseases and Related Health Problems, Tenth Revision (ICD-10) between 2016 and 2017.

### Study cohort

Among the 1,482,956 live-born infants between 2004 and 2011 in Taiwan, 4864 died before age 6 years, and 846 with any one of the following conditions that might affect neurodevelopmental outcomes were excluded: chromosome abnormalities (n = 193), brain anomalies (n = 263), and congenital infection (n = 390). To reduce the potential familial-genetic influence on ASD outcome, mothers with multiple births (n = 41,921) or more than one singleton births (n = 352,761) were excluded from analysis. Among the remaining 1082,564 mother–child pairs, after excluding 8710 with incomplete data or unidentified maternal hypertension, there were 18,131 with HDP and 1,055,723 without any maternal hypertension. To diminish the imbalance of covariates and GA-related risks of neonatal morbidities between the HDP and the normotensive group, propensity score matching was used to construct an exposure-control cohort. The propensity score, which was calculated via the logistic regression model, provided matching criteria to make similar distribution of observed baseline covariates between the HDP-exposure and normotensive-control group^32,33^. Each HDP mother–child pair was propensity-score-matched with 5 control pairs without any maternal hypertension by maternal age at delivery, mode of delivery, birth year, GA, and children’s sex. The final study cohort consisted of 18,131 HDP and 90,655 normotensive control pairs (Supplementary Fig. S1). This study was conducted in accordance with the ethical standards of Taiwan Health and Welfare Data Science Center and the Institutional Review Board of Taichung Jen-Ai Hospital in Taiwan (No. 108-83), and exempted from informed consent in case of encrypted and anonymous databases.

### Definition and classification of HDP

The HDP group was further classified into 4 subgroups, including chronic hypertension, gestational hypertension, preeclampsia, and preeclampsia with superimposed chronic hypertension, according to the guidelines from International Society for the Study of Hypertension in Pregnancy^34^. Maternal chronic hypertension referred to the occurrence of hypertension before pregnancy or during the first 20 weeks of gestation, while hypertension (> 140/90 mmHg) arising de novo at or after 20 weeks included gestational hypertension (without complications), preeclampsia (complicated with proteinuria or evidence of maternal acute kidney injury, liver dysfunction, neurological features, hemolysis or thrombocytopenia), and preeclampsia superimposed on chronic hypertension^34^. The diagnoses of HDP were identified with ICD-9 codes on at least one inpatient or at least three outpatient NHIRD records (Supplementary Table S2).

### ASD outcome and covariates

ASD was diagnosed by child psychiatrists using Diagnostic and Statistical Manual of Mental Disorders—Fourth or Fifth Edition^1,2^, or using Autism Diagnostic Observation Schedule and Autism Diagnostic Interview—Revised (Western Psychological Service, Los Angeles, CA, USA), and identified with ICD-9 or ICD-10 codes from NHIRD records of at least one hospital admission or at least three ambulatory clinic visits. The covariates that might have influence on ASD outcome were defined and recorded by ICD codes (Supplementary Table S2). Maternal factors included age at delivery, pre-pregnancy obesity, diabetes mellitus (chronic or gestational), psychiatric/mental disorders, perinatal infection, antepartum hemorrhage, and mode of delivery; while infant factors comprised GA, birth weights, SGA, sex, birth season, birth (enrollment) year, and admission to a neonatal intensive care unit within 7 days after birth. GA was further stratified into 3 groups— < 32 weeks (very preterm birth), 32–36 weeks (moderate-to-late preterm birth), and > 36 weeks (term birth). SGA was defined as birth weights lower than the 10th percentile for sex and GA according to the Fenton growth chart^35^.

### Statistical analysis

The differences in maternal and infant characteristics and the rates of ASD between the HDP and normotensive group were compared by *t* test or χ^2^ analysis^36^. The cumulative rates of ASD over time in children exposed to maternal HDP were examined using the survival or time-to-event analysis, in which the time origin was the date of childbirth. Children were followed up for the occurrence of ASD from their birthdays to 31st December 2017 when they were 6–13 years old (the end point of time). Kaplan–Meier survival curves were plotted to compare the crude cumulative incidences of ASD among the normotensive group and HDP subgroups. Using the normotensive group as the reference, the associations between HDP subgroups and the cumulative risks of ASD were assessed with hazard ratios in the Cox proportional hazards regression models^37^. Potential predictors of ASD in the univariable analyses were fitted into a multivariable model by stepwise selection of variables, with estimation of adjusted hazard ratios and 95% confidence intervals.

The potential effects of preterm birth and SGA on the association between HDP subgroups and ASD outcomes were determined using moderation and mediation analysis. Moderation, also known as effect modification, examines the interacting effect of a third variable (or moderator) on the relationship between a predictor and an outcome variable^38^, while mediation explores whether the observed relationship between a predictor and an outcome variable is transmitted through a third variable (or mediator) using a regression-with-residuals approach^39^. The sensitivity analysis of unmeasured confounding was performed using E-value estimation for the adjusted odds ratio, which denotes the degree to which one or more unobserved confounders would need to increase the risk of exposure and outcome to fully account for the observed associations^40^. All analyses were performed using the SAS v9.4 statistical software (SAS Institute, Cary, NC, USA).

### Ethics approval

The study was conducted in accordance with the Declaration of Helsinki, and was approved by the Institutional Review Board of Taichung Jen-Ai Hospital in Taiwan (No. 108-83).

### Informed consent statement

The need for informed consent was waived by the Institutional Review Board of Taichung Jen-Ai Hospital in Taiwan due to encrypted and anonymous databases without personal identification.

## Supplementary Information

Supplementary Information.

## Data Availability

The data analyzed in this study were provided from the Health and Welfare Data Science Center (HWDSC) affiliated to Ministry of Health and Welfare in Taiwan. Due to legal restrictions imposed by the Taiwan government related to the Personal Information Protection Act, the databases cannot be made publicly available. Requests for access to these data should be made to HWDSC in Taiwan (https://dep.mohw.gov.tw/dos/cp-5119-59201-113.html).
