# Supplementary material for: Preterm birth and small for gestational age potentiate the association between maternal hypertensive pregnancy and childhood autism spectrum disorder
Source: Sci Rep. 2023 Jun 13;13:9606. doi: 10.1038/s41598-023-36787-w (PMC10264454; doi:10.1038/s41598-023-36787-w)
Supplement: Supplementary file 1 — Supplementary Information. [file 41598_2023_36787_MOESM1_ESM.pdf]

Preterm birth and small for gestational age potentiate the association between maternal hypertensive pregnancy and childhood autism spectrum disorder

Lan-Wan Wang, Hung-Chih Lin, Ming-Luen Tsai, Yu-Tzu Chang, Yu-Chia Chang

Supplementary Information

Supplementary Figure S1. Flow chart of study cohort selection

Supplementary Table S1. Mediating effects of preterm birth and SGA on the association between hypertensive disorders of pregnancy and autism spectrum disorder

Supplementary Table S2. ICD-9/ICD-10 codes of variables

Supplementary Figure S1. Flow chart of study cohort selection

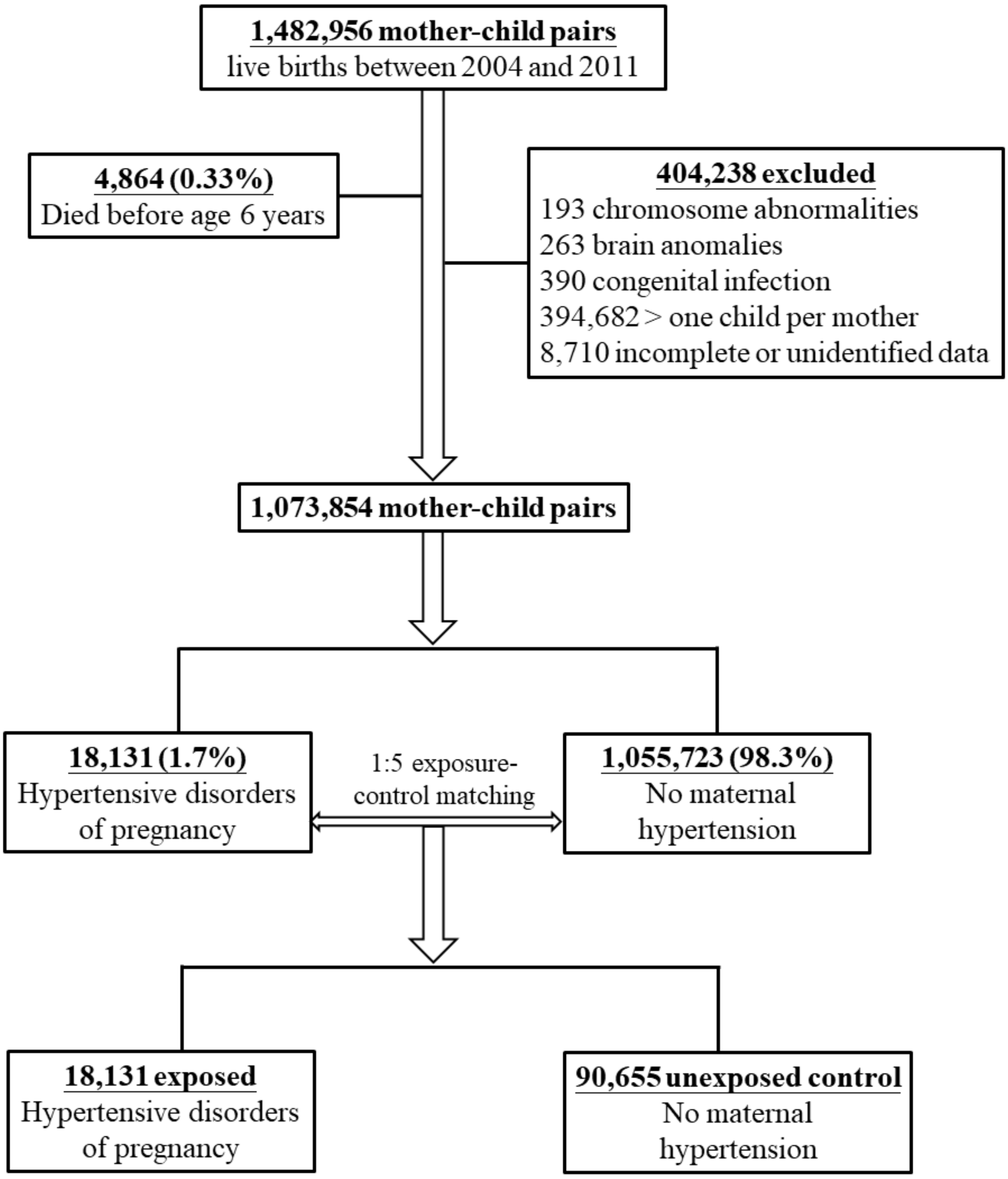

Supplementary Table S1. Mediating effects of preterm birth (GA<= 36 weeks) and SGA on the association between hypertensive disorders of pregnancy and autism spectrum disorder

	Adjusted odds ratio (95% confidence interval)			log (indirect)/log (total effect), %
	Direct effect	Indirect effect	Total effect	
**Mediator: GA ($\leq$36 vs. >36 [reference])[a]**				
Hypertensive disorders of pregnancy				
No	1.00 (reference)	1.00 (reference)	1.00 (reference)	
Chronic hypertension	1.35 (1.04-1.66)	1.00 (1.00-1.00)	1.35 (1.04-1.66)	0.34
Gestational hypertension	1.15 (0.76-1.53)	1.00 (0.98-1.01)	1.14 (0.76-1.53)	2.56
Preeclampsia	1.43 (1.18-1.68)	1.00 (1.00-1.00)	1.43 (1.18-1.68)	0.56
Preeclampsia with chronic hypertension	1.48 (0.99-1.98)	1.00 (1.00-1.00)	1.48 (0.99-1.98)	0.14
All types	1.39 (1.22-1.55)	1.00 (1.00-1.00)	1.39 (1.23-1.55)	0.17
**Mediator: SGA (Yes vs. No [reference])[b]**				
Hypertensive disorders of pregnancy				
No	1.00 (reference)	1.00 (reference)	1.00 (reference)	
Chronic hypertension	1.66 (1.20-2.12)	1.04 (0.97-1.11)	1.73 (1.27-2.19)	9.17
Gestational hypertension	1.76 (1.14-2.37)	1.03 (0.95-1.12)	1.82 (1.20-2.43)	7.36
Preeclampsia	1.46 (1.10-1.82)	1.04 (0.93-1.15)	1.52 (1.18-1.86)	11.34
Preeclampsia with chronic hypertension	0.85 (0.34-1.36)	1.04 (0.77-1.32)	0.89 (0.42-1.36)	33.38
All types	1.49 (1.26-1.72)	1.04 (0.98-1.10)	1.55 (1.32-1.77)	10.74

GA, gestational age (week); SGA, small for gestational age. [a]adjusted for maternal factors (age at delivery, pre-pregnancy obesity, diabetes mellitus, psychiatric/mental illness, perinatal infection, antepartum hemorrhage, cesarean section), and infant factors (SGA, male sex, birth season, birth year); [b]adjusted for maternal factors (age for delivery, pre-pregnancy obesity, diabetes mellitus, psychiatric/mental illness, perinatal infection, antepartum hemorrhage, cesarean section), and infant factors (GA, male sex, birth season, birth year).

Supplementary Table S2. ICD-9/ICD-10 codes of variables

Variable	ICD-9[a]	ICD-10[b]
**Hypertensive disorders of pregnancy**		
Chronic hypertension	401.0-9; 642.00-04, 10-14, 20-24	
Gestational hypertension	642.30-34	
Preeclampsia	642.40-44, 50-54, 60-64	
Preeclampsia with chronic hypertension	642.70-74	
**Neurodevelopmental outcome in offspring**		
Autism spectrum disorder	299.00, 01, 10, 11, 80, 81, 90, 91	F840, 843, 845, 848, 849
**Maternal covariates**		
Obesity	278.00, 278.01	
Diabetes mellitus	648.00-04, 80-84;	
	250.00-03, 10-13, 20-23, 30-33; 40-43, 50-53, 60-63, 70-73, 80-83, 90-93	
Psychiatric/mental illness	290-319	
Perinatal infection	659.20-23; 670.00-04; 672.00-04; 658.20-23, 40-43	
Antepartum hemorrhage	641.10-13, 20-23, 30-33, 80-83, 90-93	
Cesarean section	669.70-71	

[a]ICD-9 codes for maternal records from 2002 to 2011 and children's records from 2004 to 2015; [b]ICD-10 codes for children's records starting from 2016 in Taiwan.
